# Kidney transplantation in lupus patients: from contraindicated to an excellent option for renal function replacement

**DOI:** 10.1590/2175-8239-JBN-2025-E009en

**Published:** 2025-06-02

**Authors:** Vinicius Daher Alvares Delfino, Abel Esteves Soares

**Affiliations:** 1 Pontifícia Universidade Católica (PUC), Disciplina de Nefrologia, Londrina, PR, Brazil.; 2 Hospital Evangélico de Londrina, Londrina, PR, Brazil.

Renal involvement is a common and feared complication in individuals with systemic lupus erythematosus (SLE), affecting 30–60% of these patients. Diagnostic and therapeutic advances have improved both renal survival and the survival of patients with lupus nephritis (LN); nevertheless, 10–20% of them will require renal replacement therapy (RRT) within 10 years of LN diagnosis. For several decades, kidney transplantation (Tx) in lupus patients was viewed with skepticism and often considered contraindicated. There were concerns regarding graft loss due to recurrence of nephritis, as well as an increased risk of infection and death. This understanding began to change based on studies conducted between 1975 and 1999. In 2020, Ward, evaluating data from the United States Renal Data System (USRDS) from 1987 to 1994, compared kidney transplants in lupus patients (n = 1,160) vs. other etiologies (n = 43,156) and demonstrated that both graft and patient survival in lupus patients did not differ from that observed in patients with other underlying kidney diseases^1^. Since then, several – though not all – studies have supported these findings.

Due to the considerable heterogeneity of the studies, which were conducted at different times, across populations of different ethnicities, and with varying access to healthcare and immuno­suppressive drugs, among other particu­larities, it is essential that national studies on the subject be conducted in order to gain a more detailed understanding of the situation in Brazil. Pachi et al.^2^ studied a retrospective cohort of 99 lupus patients who underwent kidney transplantation at a single center in southern Brazil, a facility with extensive experience in transplantation, between 1997 and 2023. According to the period of immunosuppression, these patients were divided into two groups: G1 (n = 30; received kidney transplants between 1997 and 2008) and G2 (n = 69; transplanted in 2009 or later). Immunosuppression was primarily based on prednisone, cyclosporine, and azathioprine in G1, and on prednisone, tacrolimus, and mycophenolate in G2^2^.

The majority of the cohort was composed of women (87%). Two-thirds of patients were white, and the median time on dialysis and age at transplantation were 1.5 years and 30 years, respectively. Patients in G1 more frequently received a first transplant, a higher number of grafts from living donors, and had lower calculated PRA values (96.6% vs. 78.2%, P = 0.035; 50% vs. 18.8%, P = 0.003; 6.5 vs. 26.0, P = 0.039, respectively). A calculated PRA > 80% was observed in 11 patients (15.9%) in G2 and in no patients in G1. The urine protein-to-creatinine ratio (uPCR) and estimated glomerular filtration rate (eGFR) were similar between the two groups at both 1 and 5 years of follow-up. The 5-year patient survival was 100% in G1, compared with 87% in G2. Graft survival was similar between groups (G1: 86.7% vs. G2: 67.5%; P = 0.054), although a trend toward higher graft survival in G1 may be considered.

A somewhat surprising finding was the higher 10-year patient and graft survival rates in G1compared to G2 (96.2% vs. 65.3%, P = 0.018 and 66.7% vs. 37.9%, P = 0.025, respectively). An attempt was made to identify possible explanations for these differences using the Cox proportional hazards model (variables included: sex, age at transplantation, dialysis modality, donor type, calculated PRA, presence of anti-HLA antibodies, HLA-DR typing, episodes of rejection, LN recurrence, eGFR, and uPCR at the end of the first year); however, none of the tested variables could be identified. The authors argue that the number of patients who reached a follow-up period of 10 years or more post-transplant was very small, which could explain the differences in survival between the groups. Following similar reasoning, the relatively small number of patients in each group may also have represented a limitation for identifying variables possibly associated with survival in the COX model. Thus, it seems more prudent to focus comments on patient and graft survival rates 5 years after transplantation, which are similar to those reported in the current literature^1,3,4,5^ and in previous national studies^6,7^.

The effort and dedication of the authors, the organization of the service, and the importance of the study findings deserve full recognition. An important finding was the low recurrence of LN in the transplanted kidney (5%) with no graft loss, similar to what is currently described in the literature^8^. Another finding was that the 5-year kidney graft survival among the 99 patients studied did not differ from that of patients transplanted for other underlying kidney diseases (73.6% vs. 71.5%, respectively, P = 0.308). Studies do not always report similar survival rates for patients and lupus kidney grafts when compared to those of recipients with other kidney diseases. For example, Jiang et al.^4^ found lower survival rates among lupus transplant recipients in the Australia and New Zealand Transplant Registry, a finding similar to that reported by Derner et al.^3^ following analysis of kidney transplants recorded in the European Renal Association (ERA) Registry between 1992 and 2016^9^. As further evidence of the complexity of this issue, Jiang et al.^4^, in a recent meta-analysis of studies with a larger number of lupus patients, reported that patient survival is lower in case-control studies but not in clinical trials. Conversely, lower kidney graft survival is observed in clinical trials, but not in case-control studies.

The Gordian knot of the issue seems to be recognizing which RRT modality provides the longest survival for lupus patients. Recently, Brilland et al.^5^ conducted an elegant analysis of the survival of lupus patients who were on the waiting list for a deceased-donor kidney transplant between 2002 and 2022, using data from the French Renal Epidemiology and Information Network (REIN). Of the 882 patients who initiated RRT, 636 (72%) were included in the waiting list and 470 (74%) received a kidney transplant. After a median of 80 months, a 60% reduction in the risk of death was observed among transplant recipients compared to those who remained on dialysis (*hazard ratio* [HR]: 0.40 [95% CI]; 0.240–0.67, P < 0.001). The 10-year patient survival was 83% for transplant recipients and 60% for non-transplanted patients (P < 0.001). Sensitivity analyses, excluding recipients of living-donor transplants and individuals removed from the waiting list, did not alter the overall picture^5^.

**Figure 1:**
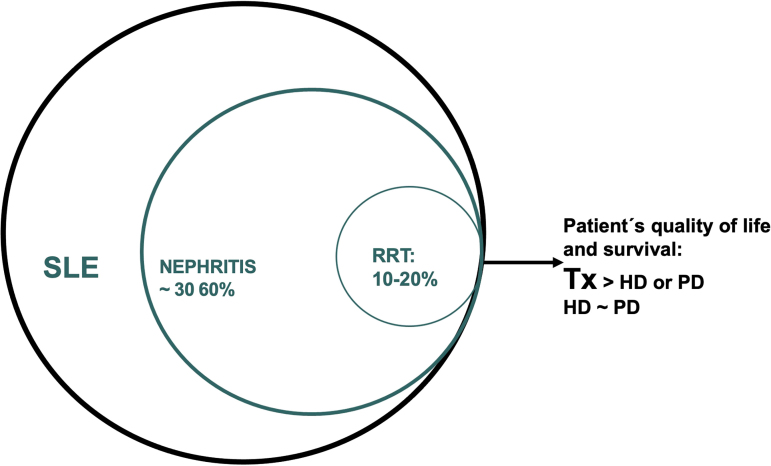
Approximate percentage of lupus patients presenting with nephritis, who require RRT, and comparison of different RRT regarding survival of these patients.

Most available studies indicate that survival rates for lupus patients on hemodialysis and peritoneal dialysis are similar, but lower than those observed in kidney transplantation. Added to this is the higher quality of life in the transplant group. The KDIGO 2024 Clinical Practice Guidelines for the Management of Lupus Nephritis recommend kidney transplantation as the preferred modality of RRT for the long-term treatment of these patients. The guidelines further recommend that transplantation be performed as soon as SLE is quiescent, since the results of the procedure are better in patients who remain on dialysis for shorter periods of time^10^. Figure 1 depicts a simplified view of the onset and course of nephritis in lupus patients, as well as the available RRT modalities. Considering the aforementioned, one possible way to further expand knowledge in Brazil about transplantation in lupus patients would be to establish a national registry on the subject. Both lupus patients and transplant centers could benefit from such a registry in their decision-making processes.

Abbreviations – SLE: systemic lupus erythematosus; RRT: renal replacement therapy; Tx: transplant; HD: hemodialysis; PD: peritoneal dialysis.
