# Supplementary material for: Kidney transplantation in lupus patients: from contraindicated to an excellent option for renal function replacement
Source: J Bras Nefrol. 2025 Jun 2;47(2):e2025E009. doi: 10.1590/2175-8239-JBN-2025-E009en (PMC12129480; doi:10.1590/2175-8239-JBN-2025-E009en)
Supplement: Translation

# Translation: Transplante renal no paciente lúpico: de contraindicado a excelente opção de reposição da função renal

**DOI:** 10.1590/2175-8239-JBN-2025-E009pt

**Authors:** Vinicius Daher Alvares Delfino, Abel Esteves Soares

**Affiliations:** 1 Pontifícia Universidade Católica (PUC), Disciplina de Nefrologia, Londrina, PR, Brasil.; 2 Hospital Evangélico de Londrina, Londrina, PR, Brasil.

O acometimento renal é uma complicação frequente e temida nos indivíduos com lúpus eritematoso sistêmico (LES), acometendo de 30–60% desses pacientes. Avanços diagnósticos e terapêuticos melhoraram a sobrevida renal, bem como a sobrevida dos pacientes com nefrite lúpica (NL), mas, ainda assim, 10–20% deles necessitarão de terapia renal substitutiva (TRS) após 10 anos do diagnóstico de NL. O transplante (Tx) renal para o paciente lúpico foi, por algumas décadas, visto com ceticismo e, não raramente, considerado contraindicado. Havia receio de perda do enxerto por recorrência da nefrite, além de maior risco de infecções e de óbito. Esse entendimento começou a mudar a partir de estudos realizados entre 1975 e 1999. Em 2020, Ward, avaliando dados do *United States Renal Data System* (USRDS) de 1987 a 1994, comparou Txs renais em pacientes lúpicos (n = 1.160) vs. outras etiologias (n = 43.156) e demonstrou que a sobrevida dos enxertos e a dos pacientes lúpicos não diferia da observada em pacientes com outras doenças renais de base^1^. Desde então, diversos estudos – embora nem todos – vêm comprovando esses achados.

Devido à grande heterogeneidade dos estudos, realizados em diferentes períodos, em populações de distintas etnias e com variações no acesso à saúde e aos medicamentos imunossupressores, entre outras particularidades, é fundamental que haja estudos nacionais sobre o tema, a fim de permitir um conhecimento mais detalhado dessa realidade no país. Pachi et al.^2^, estudaram uma coorte retrospectiva de 99 pacientes lúpicos que receberam Tx renal em uma única instituição no sul do Brasil, com ampla experiência em transplantação, entre 1997 e 2023. De acordo com o período de imunossupressão, esses pacientes foram divididos em dois grupos: G1 (n = 30; receberam Txs renais entre 1997 e 2008) e G2 (n = 69; transplantados a partir de 2009, inclusive). A imunossupressão dos pacientes foi baseada principalmente em prednisona, ciclosporina e azatioprina no G1, e em prednisona, tacrolimo e micofenolato no G2^2^.

A maioria da coorte foi composta por mulheres (87%). Dois terços dos pacientes eram brancos e as medianas do tempo em diálise e da idade ao transplante foram de 1,5 ano e 30 anos, respectivamente. Os pacientes do G1 receberam, mais frequentemente, um primeiro Tx, um número maior de enxertos de doadores vivos e apresentaram PRA calculado mais baixo (96,6% vs. 78,2%, P = 0,035; 50% vs. 18,8%, P = 0,003; 6,5 vs. 26,0, P = 0,039, respectivamente). Um PRA calculado > 80% esteve presente em 11 pacientes (15,9%) do G2 e em nenhum paciente do G1. A razão proteína/creatinina urinária (RPCU) e a taxa de filtração glomerular estimada (TFGe) foram semelhantes com 1 e 5 anos de seguimento entre os dois grupos. A sobrevida em 5 anos dos pacientes no G1 foi de 100%, em comparação a 87% no G2. A sobrevida dos enxertos foi semelhante entre os grupos (G1: 86,7% vs. G2: 67,5%; P = 0,054), embora se possa considerar uma tendência à maior sobrevida dos enxertos no G1.

Um tanto quanto surpreendente foi o achado de maior sobrevida de pacientes e enxertos aos 10 anos no G1 em comparação ao G2 (96,2% *vs.* 65,3%, P = 0,018 e 66,7% *vs.* 37,9%, P = 0,025, respectivamente). Buscou-se identificar eventuais explicações para essas diferenças utilizando o modelo de risco proporcional de Cox (variáveis incluídas: sexo, idade ao Tx, tipo de diálise, tipo de doador, PRA calculado, presença de anticorpos anti-HLA, tipagem HLA-DR, episódios de rejeição, recorrência de NL, TGFe e RPCU ao final do primeiro ano); contudo, nenhuma variável dentre as testadas pôde ser identificada. Os autores argumentam que o número de pacientes que alcançaram um período de seguimento de 10 anos ou mais pós-Tx foi muito pequeno, o que poderia explicar as diferenças nas sobrevidas entre os grupos. Seguindo um raciocínio semelhante, o número relativamente reduzido de pacientes em cada um dos grupos pode também ter representado uma limitação para a identificação, no modelo de COX, de variáveis possivelmente associadas às sobrevidas. Dessa maneira, parece mais prudente concentrar os comentários nas sobrevidas de pacientes e enxertos após 5 anos de transplante, as quais se mostram semelhantes às relatadas na literatura contemporânea^1,3-5^ e em estudos nacionais anteriores^6,7^.

O esforço e o desvelo dos autores, a organização do serviço, bem como a importância dos achados do estudo merecem pleno reconhecimento. Um achado importante foi a baixa recorrência da NL no rim transplantado (5%), sem acarretar perda do enxerto, dado semelhante ao atualmente descrito na literatura^8^. Outro achado foi a constatação de que a sobrevida do enxerto renal em 5 anos, entre os 99 pacientes estudados, não diferiu da sobrevida dos enxertos de pacientes transplantados por outras doenças renais de base (73,6% vs. 71,5% respectivamente, P = 0,308). Nem sempre os estudos relatam sobrevidas semelhantes de pacientes e enxertos renais lúpicos quando comparados àquelas de receptores com outras doenças renais. Por exemplo, Jiang et al.^4^ encontraram, no Registro Austrália-Nova Zelândia de Transplantes, menor sobrevida entre receptores lúpicos, achado semelhante ao reportado por Derner et al.^3^ após análise dos Txs renais registrados no *European Renal Association Registry* (registro ERA) entre 1992 e 2016^9^. Prova de que se trata de um tema complexo, Jiang et al.^4^, em metanálise recente de estudos com maior número de pacientes lúpicos, referem que a sobrevida dos pacientes lúpicos é inferior nos estudos caso-controle, mas não nos ensaios clínicos. Por outro lado, a menor sobrevida de enxertos renais é vista nos ensaios clínicos, mas não nos estudos caso-controle.

O nó górdio da questão parece ser reconhecer qual forma de TRS proporciona maior sobrevida ao paciente lúpico. Recentemente, Brilland et al.^5^, utilizando os registros do *French Renal Epidemiology and Information Network* (REIN), analisaram de maneira muito elegante a sobrevida de indivíduos lúpicos que foram colocados em lista de espera para Tx renal de doador falecido entre 2002 e 2022. Dos 882 pacientes que iniciaram TRS, 636 (72%) foram incluídos na lista de espera e 470 (74%) receberam um Tx renal. Após uma mediana de 80 meses, observou-se uma redução de 60% no risco de óbito entre os transplantados em comparação aos que permaneceram em diálise (*hazard ratio* [HR]: 0,40 [IC 95%]: 0,240–0,67, P < 0, 001). A sobrevida dos pacientes aos 10 anos foi de 83% para os transplantados e 60% para os não transplantados (P < 0,001). Análises de Sensibilidade, com a exclusão dos receptores de Txs de doadores vivos e dos indivíduos retirados da lista de espera, não alteraram o panorama^5^.

A maioria dos estudos disponíveis indica que as sobrevidas dos pacientes lúpicos em hemodiálise e em diálise peritoneal são semelhantes entre si, porém inferiores às observadas no Tx renal. Soma-se a isso a maior qualidade de vida no grupo transplantado. O KDIGO 2024 *Clinical Practice Guidelines for the Management of Lupus Nephritis* recomenda que o Tx renal seja a modalidade de TRS preferida para o tratamento de longo prazo desses pacientes. Recomenda, ainda, que o Tx seja realizado tão logo o LES esteja quiescente, pois os resultados do procedimento são melhores naqueles pacientes que permanecem por menos tempo em diálise^10^. A Figura 1 apresenta uma visão simplificada do aparecimento e do curso da nefrite nos pacientes lúpicos, assim como das modalidades de TRS disponíveis. Diante do exposto, uma possiblidade de expandir ainda mais o conhecimento no Brasil sobre o transplante em pacientes lúpicos seria a criação de um registro nacional sobre o tema. Tanto os pacientes lúpicos quanto os serviços de transplante poderiam se beneficiar de tal registro em suas tomadas de decisão.

**Figura 1:** Porcentagem aproximada de pacientes lúpicos que apresentam nefrite, que necessitam de TRS e comparação das diferentes terapias de reposição renal quanto à sobrevida desses pacientes.

Abreviações – LES: lúpus eritematoso sistêmico; TRS: terapia renal substitutiva; Tx: transplante; HD: hemodiálise; DP: diálise peritoneal.
